# *Ophiorrhiza xinchengensis* (Rubiaceae), a new species from Guangxi, China

**DOI:** 10.3897/phytokeys.278.194094

**Published:** 2026-07-24

**Authors:** Gui-Yuan Wei, Jin-Yue Li, Rong Chen, You Nong, Qi-Min Hu, Ying-Jing Li, Xing-Yun Ji, Chuan-Gui Xu, Yun-Kai Nong

**Affiliations:** 1 Guangxi Key Laboratory of Traditional Chinese Medicine Quality Standards; Guangxi Institute of Chinese Medicine & Pharmaceutical Science, No. 20-1 Dongge Road, Nanning, Guangxi, China University of Chinese Academy of Sciences Beijing China; 2 University of Chinese Academy of Sciences, Huairou District, Beijing 101408, China Guangxi Key Laboratory of Traditional Chinese Medicine Quality Standards; Guangxi Institute of Chinese Medicine & Pharmaceutical Science Nanning China

**Keywords:** Karst, new species, *
Ophiorrhiza
*, *
Spiradiclis
*, taxonomy

## Abstract

*Ophiorrhiza xinchengensis* (Rubiaceae), a new species from the middle part of Guangxi, China, is described and illustrated on the basis of morphological evidence. This new species resembles *Ophiorrhiza longanensis* in its densely pubescent stems, linear stipules, terminal or upper axillary inflorescences, but it can be distinguished by its elliptic or oblanceolate leaf blade, base attenuate (vs. oval, ovate to broadly ovate, base cuneate), its 2–3 mm long stipules (vs. 3.8–6.5 mm long), its 3–5 mm long bracteoles (vs. 6–10 mm long) its 10–12 mm long corolla tubes (vs. 2–3 mm long) and its filaments adnate at the middle of corolla tube, styles 12–15 mm long (vs. adnate to the base of corolla tube and styles ca. 3 mm long). The new species is currently known from a single population with approximately 60–100 mature individuals. Following IUCN Criteria, it is provisionally assessed as Critically Endangered. Photographs, an illustration and a distribution map are also provided.

## Introduction

*Ophiorrhiza* L. (Linnaeus 1753) is a genus within the Rubiaceae family that is rich in species and taxonomically complex. This genus comprises species that are predominantly annual or perennial herbs, with sub-shrubs being a rare occurrence. *Ophiorrhiza* species can be readily distinguished by their obcordate (heart-shaped with the point at the top) and fattened fruits, which split open along a transverse slit at the apex into two valves (Darwin 1976; Lo 1990, 1999; Chen and Taylor 2011a; Wu et al. 2019).

The genus *Spiradiclis* Blume, belonging to the tribe Ophiorrhizeae within the Rubiaceae family, consists of roughly 60 species. These species are predominantly found in the subtropical and tropical areas of Southeast Asia, with a notable abundance in the karst terrains of southern China and northern Vietnam (Wang 2016; Zhang et al. 2018; Pan et al. 2019; Tong et al. 2020; Cai et al. 2022). Razafimandimbison and Rydin (2019) proposed that *Spiradiclis* should be regarded as a synonym of *Ophiorrhiza* L., based on molecular phylogenetic analyses and subsequent taxonomic transfers have been made (Idrees et al. 2023). Although morphological distinctions, such as linear-oblong or subglobose capsules with four valves in *Spiradiclis* (vs. contrasting with the obcordate and compressed capsules with two valves of *Ophiorrhiza*), have historically supported generic separation and there were also some describing new species in the synonymised genus *Spiradiclis* recently in China (Cai et al. 2022; Nong et al. 2025; Xiong et al. 2025; Xu et al. 2025), but this also leads to unnecessary subsequent transfer and recombination, we believe, nevertheless, that the boundaries and relationships between these two genera require further investigation. These contrasting viewpoints reflect a classic conflict in modern taxonomy: between the integration of molecular evidence and the retention of morphologically coherent taxonomic concepts that facilitate identification in the field. We here adopt the more widely accepted circumscription following molecular evidence. Accordingly, we describe the new species under *Ophiorrhiza*.

During our field surveys in Xincheng County, Guangxi Zhuang Autonomous Region in May 2025, we discovered a special fruiting *Ophiorrhiza* species growing in a karst cave. These special plants resemble *Spiradiclis hechiensis* C.Xiong & L.Wu in its densely villous stems, terminal inflorescences and colour of the corolla, but it can be distinguished by its stipules linear, 2–3 mm long (vs. broad triangular, ca. 1 mm long) and its capsules densely pubescent (vs. densely villous). After consulting relevant literature (Wang 2016; Zhang et al. 2018; Pan et al. 2019; Tong et al. 2020; Cai et al. 2022; Nong et al. 2025; Xiong et al. 2025; Xu et al. 2025), checking relevant specimens and a collection conducted recently in February in 2026 when it was flowering, we confirmed that this unusual plant is new to science and is described below.

## Materials and methods

### Morphology

The new species was described, based on field observations that were made in January 2026 and examination of herbarium specimens at GXMI. Other related *Spiradiclis* species were examined, based on online images from the Kew Herbarium Catalogue (http://apps.kew.org/herbcat/gotoHomePage.do) and JSTOR Global Plants (http://plants.jstor.org/).

Measurements were made with a tape measure and callipers. The structure of the indumentum and its distribution were observed and described under a dissecting microscope at magnifications of more than 20×.

### Sampling, DNA extraction and sequencing

In this study, total genomic DNA was extracted from silica-dried leaves using a modified cetyltrimethylammonium bromide (CTAB) protocol (Doyle and Doyle 1987) and quality was assessed by agarose gel electrophoresis. Total DNA was sent to TSINGK Biological Technology Company (Guangzhou, China) for Sanger sequencing (Sanger and Coulson 1975). The sequences of an additional 43 species were downloaded from the NCBI database. The specimen information of samples and GenBank accession numbers for all sequences are listed in Table 1.

**Table 1.** Voucher information for phylogenetic analyses and GenBank accession numbers.

**Taxa**	**Voucher**	**ITS**
*Coptophyllum bracteatum*	cz31	MH626791
*Coptophyllum bracteatum*	cz32	MH626792
*Ophiorrhiza* sp.	cz19	MH626835
*Ophiorrhiza baviensis*	az37	MH626804
*Ophiorrhiza hayatana*	cz08	MH626810
*Ophiorrhiza japonica*	az05	MH626811
*Ophiorrhiza kwangsiensis*	ba56	MH626812
*Ophiorrhiza amplifolia*	cz03	MH626801
*Ophiorrhiza* sp.	cz18	MH626834
*Ophiorrhiza* sp.	cz17	MH626833
*Keenania ophiorrhizoides*	cx21	MH626793
*Keenania tonkinensis*	cy100	MH626794
*Spiradiclis cylindrica*	az17	MH626844
*Ophiorrhiza brunonis*	cz06	MH626805
*Ophiorrhiza pedunculata*	az28	MH626823
*Ophiorrhiza angkae*	az31	MH626802
*Ophiorrhiza pseudofasciculata*	az32	MH626827
*Ophiorrhiza villosa*	az30	MH626841
*Ophiorrhiza communis*	ba44	MH626806
*Ophiorrhiza havilandii*	ba86	MH626810
*Ophiorrhiza larseniorum*	az36	MH626813
*Ophiorrhiza longiflora*	ba01	MH626819
*Ophiorrhiza ripicola*	az29	MH626830
*Ophiorrhiza subrubescens*	az35	MH626837
*Ophiorrhiza venosa*	cz22	MH626840
*Ophiorrhiza winkleri*	ba43	MH626842
*Spiradiclis bifida*	ax48	MH626843
*Ophiorrhiza fasciculata*	ba20	MH626807
*Ophiorrhiza thomsonii*	ba11	MH626838
*Ophiorrhiza harrisiana*	cz07	MH626808
*Ophiorrhiza rugosa*	ay11	MH626831
*Ophiorrhiza* sp.	ba10	MH626821
*Ophiorrhiza sanguinea*	cz16	MH626832
*Ophiorrhiza pumila*	ay18	MH626828
*Ophiorrhiza pumila*	az100	MH626829
*Ophiorrhiza peploides*	ax38	MH626824
*Ophiorrhiza leptantha*	ay16	MH626817
*Ophiorrhiza leptantha*	cz10	MH626818
*Ophiorrhiza peploides*	cz15	MH626826
*Ophiorrhiza laxa*	ax34	MH626814
*Ophiorrhiza laxa*	ay15	MH626815
*Ophiorrhiza leptantha*	ax35	MH626816
*Ophiorrhiza peploides*	ay17	MH626825
*Ophiorrhiza bibracteata*	NY2026020501	PZ594848
*Ophiorrhiza xinchengensis*	WGY2026020101	PZ594847

A total of 45 sequences (ITS) were used for molecular analysis, of which two were newly generated. All sequences were aligned using MAFFT v.7.490 (Katoh and Standley 2013). The sequences after alignment were connected in series using the ‘Concatenate’ tool in Genious Prime v.2021.1.1 (Kearse et al. 2012). Maximum Likelihood (ML) phylogenetic trees and Bayesian tree were constructed using IQ-TREE v.1.6.12 (Nguyen et al. 2015), implemented in the PhyloSuite v.1.2.3 platform (Zhang et al. 2020). Based on ModelFinder (Kalyaanamoorthy et al. 2017), the optimal substitution models were automatically selected as SYM+I+G4 under the Bayesian Information Criterion (BIC) and GTR+F+I+G4 under the Akaike Information Criterion (AIC). Nodal support was evaluated using 1000 bootstrap pseudo-replicates.

## Results and discussion

The Maximum Likelihood tree (ML) and the Bayesian phylogenetic trees (BI) recovered a strongly-supported monophyletic Ornithoboea (BS = 100%, PP = 1.00) across to the datasets, with the ingroup accessions clearly separated from the outgroup *Coptophyllum bracteatum* Korth.

Based on the ITS molecular phylogeny, the newly-proposed species *Ophiorrhiza xinchengensis* demonstrates a distinct phylogenetic affinity that firmly supports its recognition as an independent taxon, it was positioned in close proximity to *O. havilandii* and *O. hayatana*, yet clearly separated as an individual terminal branch (BS = 95%, PP = 0.97). This exclusive branching pattern indicates that it possesses a unique evolutionary trajectory independent of its morphologically similar congeners.

The species of *Ophiorrhiza* do not form a monophyletic group; instead, they are intermingled with representatives of *Keenania*, *Spiradiclis* and *Coptophyllum*. This suggests that, based on the ITS marker, the generic boundaries between *Ophiorrhiza* and its allied genera remain unclear and integrative evidence from morphology and broader population sampling is required to re-assess the taxonomic delimitation of these groups.

### Taxonomic treatment

#### Ophiorrhiza xinchengensis

Taxon classificationPlantaeGentianalesRubiaceae

Y.Nong & G.Y.Wei sp. nov.

02EA8B4B-B004-54C5-9CD9-BBC0D656A3F8

urn:lsid:ipni.org:names:77387680-1

Figs 1, 2, 3, 4

##### Chinese name.

“xīn chéng luó xù cǎo” (忻城螺序草).

**Figure 1.**
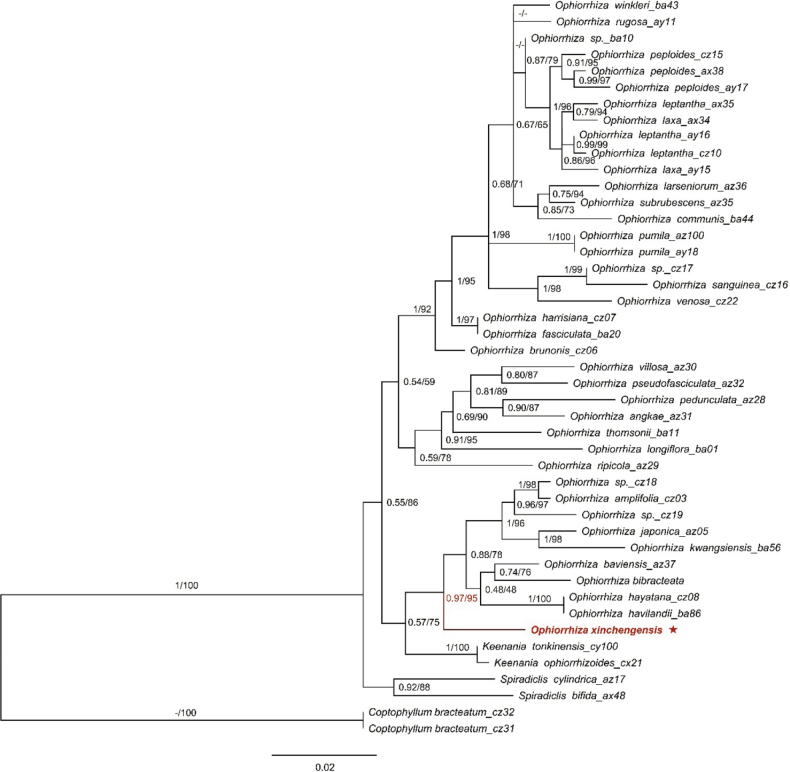
Maximum Likelihood tree (ML) and Bayesian phylogenetic trees (BI) from analysis of the ITS sequence. The posterior probabilities (PP) of BI and bootstrap percentages (BS) of ML are listed at each node (only shown if BS ≥ 50% or PP ≥ 0.50). The new species is highlighted in red.

**Figure 2.**
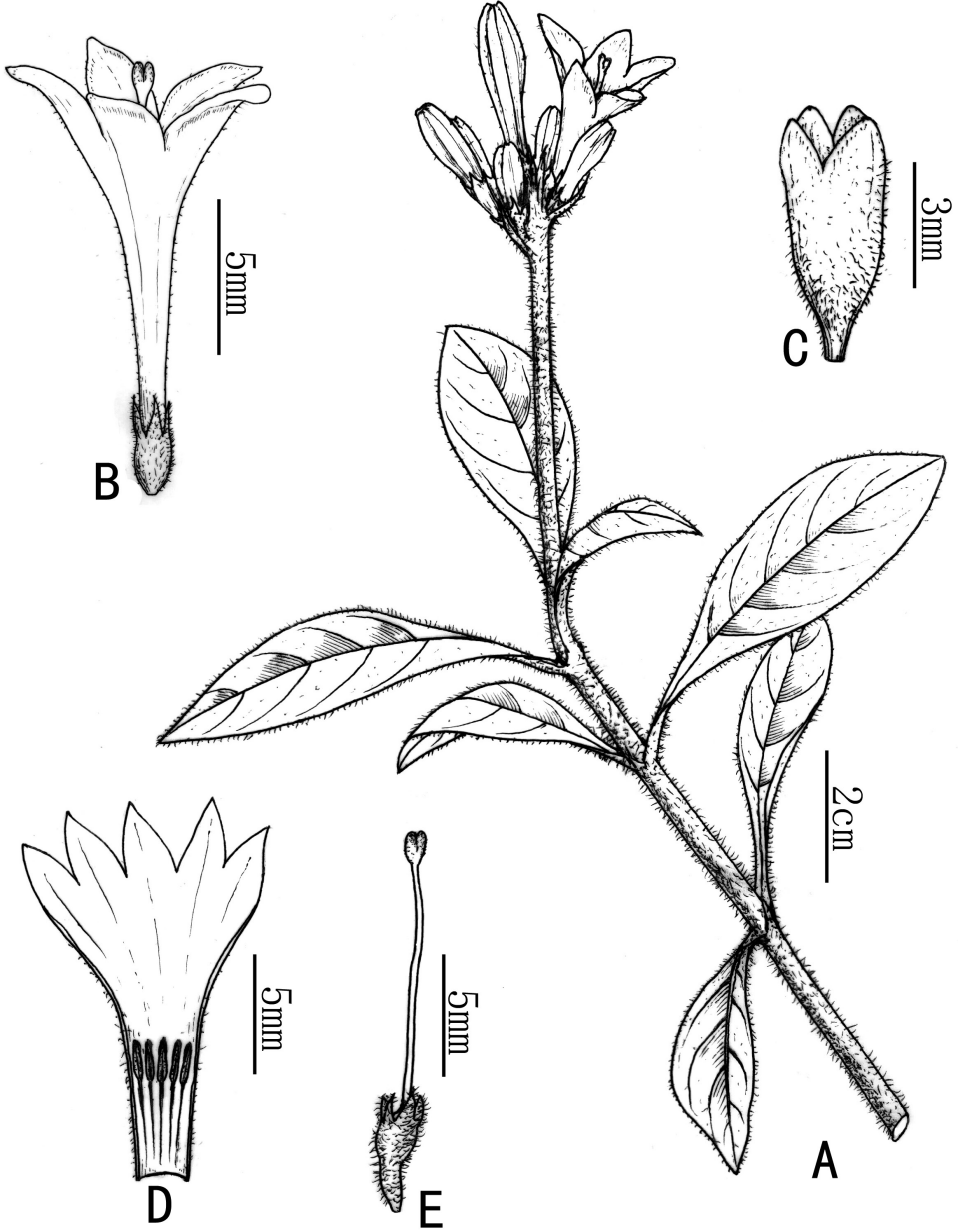
Line drawing of *Ophiorrhiza xinchengensis* Y.Nong & G.Y.Wei. **A**. Flowering branch; **B**. Longistylous flower (lateral view); **C**. Capsules (lateral view); **D**. Dissected longistylous flower (showing stamens borne at the middle of the corolla tube); **E**. Hypanthium, calyx, style and stigma (drawn by Xin-Cheng Qu).

**Figure 3.**
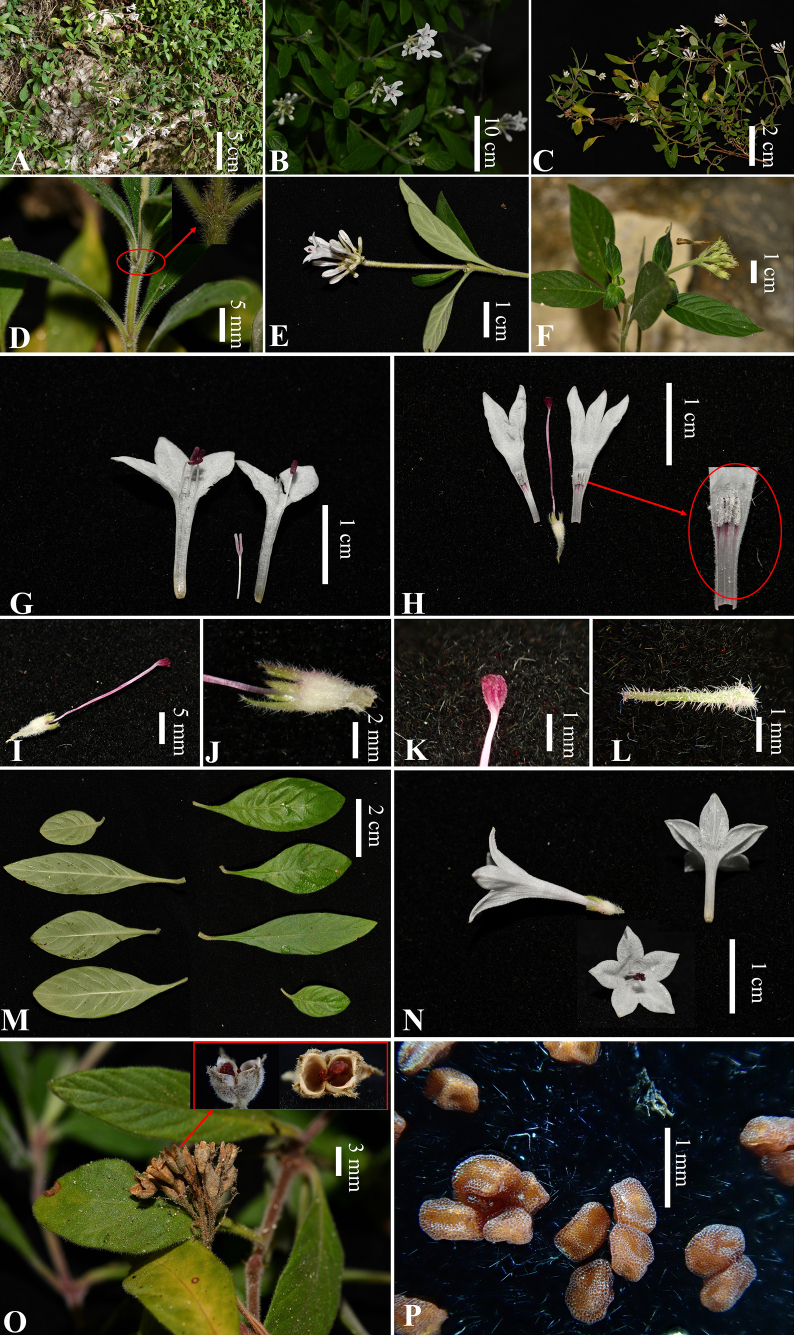
*Ophiorrhiza xinchengensis* Y.Nong & G.Y.Wei. **A**. Habitat; **B, C**. Flowering plant; **D**. Stem, showing the linear stipules; **E**. Inflorescences; **F**. Fruiting plant; **G**. Dissected brevistylous flower; **H**. Dissected longistylous flower; **I**. Hypanthium, calyx, style and stigma; **J**. Ovary and calyx; **K**. Stigma; **L**. Bracts; **M, N**. Leaf (abaxially and adaxially); **O**. Capsule; **P**. Seeds.

**Figure 4.**
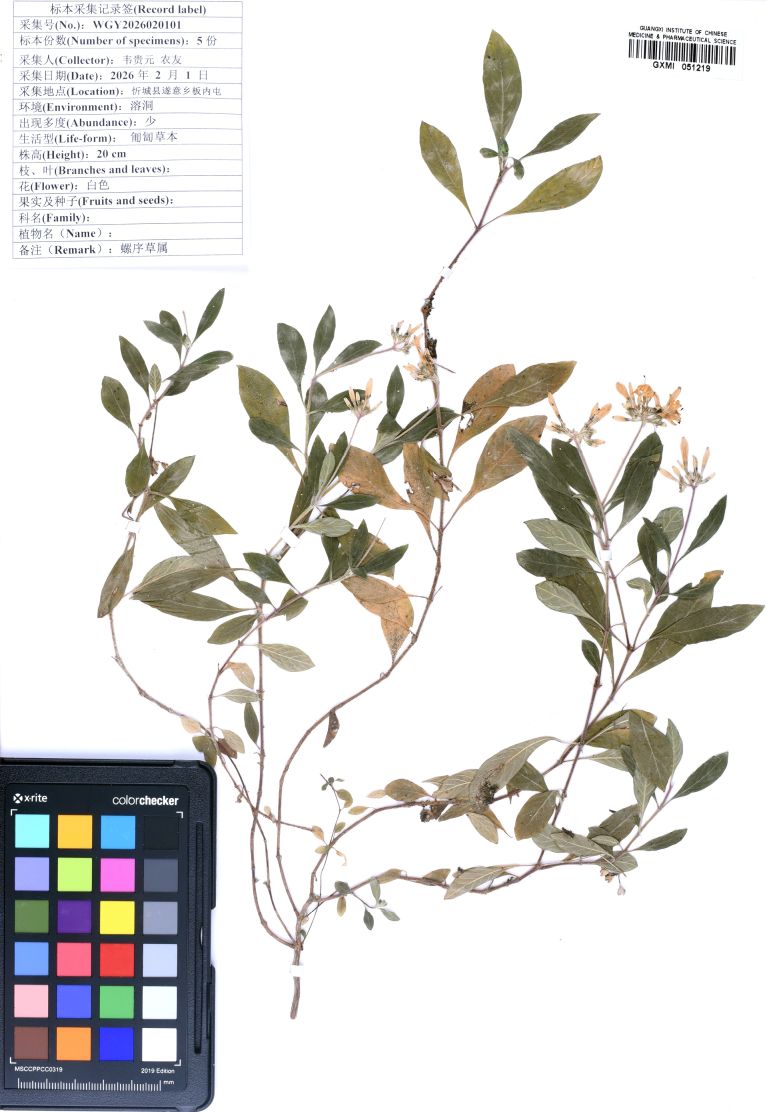
Type specimen of *Ophiorrhiza xinchengensis* Y.Nong & G.Y.Wei.

##### Diagnosis.

*Ophiorrhiza xinchengensis* is resembles *Ophiorrhiza longanensis* (R.J.Wang) Razafim. & Rydin in its densely pubescent stems, linear stipules, terminal or upper axillary inflorescences, but it can be distinguished by its elliptic or oblanceolate leaf blade, base attenuate (vs. oval, ovate to broadly ovate, base cuneate), its 2–3 mm long stipules (vs. 3.8–6.5 mm long), its 3–5 mm long bracteoles (vs. 6–10 mm long), its 10–12 mm long corolla tubes (vs. 2–3 mm long) and its filaments adnate at the middle of corolla tube, styles 12–15 mm long (vs. adnate to the base of corolla tube, styles ca. 3 mm long).

##### Type.

China • Guangxi: Xincheng County; 25°28'N, 108°53'E, alt. 400 m; 1 February 2026; *Gui-Yuan Wei WGY2026020101* (GXMI). (Holotype: 051219·GXMI!; isotypes: IBK!).

##### Description.

Perennial herbs, decumbent, not rooting at nodes; stem terete, densely villous; stipules linear, 1–2 mm long, densely villous; petiole 5–10 mm, densely villous; leaf blade drying papery, elliptic or oblanceolate, 2–5 × 1–1.8 cm, densely pubescent adaxially and abaxially, base attenuate, apex obtuse to rounded; secondary veins 5–7 pairs. Stipules linear, densely pubescent, 2–3 mm long. Inflorescences terminal or upper axillary, corymbose-cymose, 6–15 flowered; peduncle 3–5 cm, pubescent; bracteoles linear, 3–5 mm long, densely pubescent; pedicels 1–2 mm, pubescent. Flowers distylous. Hypanthium obconical, 1–2 mm long, pubescent; lobes 5, linear-lanceolate, 1.5–2 mm long, pubescent. Corolla funnel-form, white, sparsely pubescent outside, tubes 10–12 mm long, ca. 1 mm wide; ovary 2-celled, ovules on the axile placentas. Longistylous flower: corolla tube with a white pubescent ring of long hairs near the middle of the inside and sparsely pubescent above middle; lobes subovate, 5–6 × 4–5 mm, glabrous inside; stamens 5; anthers oblong-linear, ca. 1.5 mm long; stamens below the pubescent ring and included, filaments adnate at the middle of corolla tube, ca. 1 mm long; styles exserted, 12–15 mm long, glabrous, stigma lobes capitate, lobes 1–2 mm long. Brevistylous flower: corolla tube sparsely pubescent outside and densely pubescent inside; lobes subovate, 5–6 × 4–5 mm, glabrous inside; stamens 5; anthers oblong-linear, ca. 3 mm long; stamens exserted, filaments adnate at the throat of corolla tube, 4–5 mm long; styles 5–6 mm long, glabrous, stigma lobes capitate, lobes 2–3 mm long. Capsules ellipsoid, 2–3 mm long, densely pubescent, valves 4. Seeds numerous, angular, with reticulate patterns on the surface, ca. 0.2 mm in diam.

##### Etymology.

The specific epithet “xinchengensis” refers to the type location of the new species.

##### Distribution and habit.

**Figure 5.**
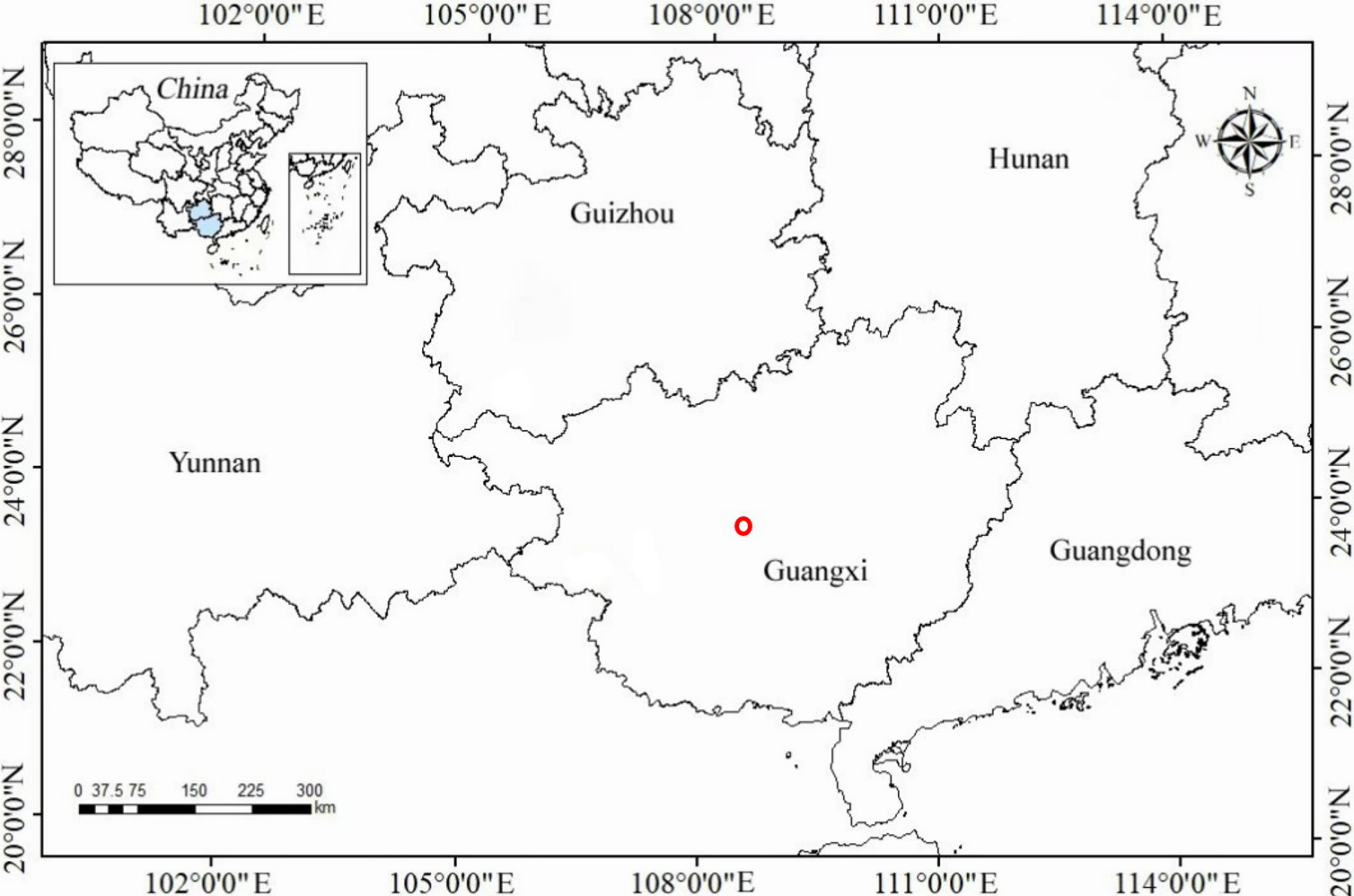
Type locality of *Ophiorrhiza xinchengensis* Y.Nong & G.Y.Wei (red circle) in Guangxi, China.

Currently, *Ophiorrhiza xinchengensis* is known only from the middle of Guangxi, China (Fig. 5). It has been mainly found growing in a karst cave at an elevation of about 400 m.

##### IUCN red list category.

Based on our field surveys conducted in 2025–2026, we estimated the single known population of the new species to contain approximately 60–100 mature individuals. The extent of occurrence (EOO) is ca. 200 m^2^ and the area of occupancy (AOO) is ca. 30 m^2^. Following the IUCN Red List Categories and Criteria (IUCN 2024), we provisionally assess the species as Critically Endangered [CR B1+2ab(iii)] because of its extremely restricted distribution, single location and potential habitat degradation.

##### Additional specimen.

Xincheng • Southeast Guangxi: karst cave, 20 May 2025, fr. *Y Nong NY2025052001* (GXMI!).

## Discussion

*Ophiorrhiza xinchengensis* is similar to *Ophiorrhiza scabrida* (D.Fang & D.H.Qin) Razafim. & Rydin, but it differs in its elliptic or oblanceolate leaf blade, base attenuate (vs. ovate, narrowly ovate, or lanceolate, base obtuse), its stipules linear, 2–3 mm long, densely pubescent (vs. subtriangular, 0.7–1 mm, subglabrous), its corolla white, sparsely pubescent abaxially, tubes 10–12 mm long (vs. white sometimes flushed with pink or purple, glabrous inside and outside; tube 25–26 mm long) and its filaments adnate at the middle of corolla tube, styles 12–15 mm long (vs. adnate to the throat of corolla tube, styles ca. 19 mm long).

*Ophiorrhiza xinchengensis* is similar to *Spiradiclis hechiensis* C.Xiong & L.Wu, but it can be distinguished by its linear stipules, 2–3 mm long (vs. broad triangular, ca. 1 mm long), its straight corolla (vs. constricted at the throat), its longer corolla tubes 10–12 mm long (vs. 6–8 mm long), its corolla tube with a white pubescent ring of long hairs near middle inside (vs. densely yellow villous inside the throat), its glabrous corolla lobes inside (vs. glandular-pubescent) and its longer styles, 12–15 mm long (vs. 4.5–5 mm long). Morphological differences amongst similar species are shown in Table 2.

**Table 2.** Main morphological differences amongst *Ophiorrhiza xinchengensis*, *Spiradiclis hechiensis*, *O. longanensis* and *O. scabrida* (Chen and Taylor 2011b; Wen et al. 2015; Xiong et al. 2025).

**Morphological traits**	***O. xinchengensis***	***S. hechiensis***	***O. longanensis***	***O. scabrida***
Leaves	elliptic or oblanceolate, 2–5 × 1–1.8 cm, base attenuate	obovate to rhombic-elliptic, 0.8–3.6 × 0.4–1.6 cm, base attenuate to narrowly cuneate	oval, ovate to broadly ovate, 1.1–5 (–6.2) × 0.5–3 cm, base cuneate	ovate, narrowly ovate, or lanceolate 2–9 × 1–3.3 cm, base obtuse
Petiole	5–10 mm, densely villous	0–5 mm long, villous	0.5–2 cm long, sparsely pubescent	2–5 mm, glabrous or usually pubescent
Stipules	linear, 2–3 mm long, densely pubescent	broad triangular, ca. 1 mm long, pubescent	linear, 3.8–6.5 mm long, pubescent	subtriangular, 0.7–1 mm, subglabrous
Inflorescence	6–15 flowered	1–4-flowered, sometimes up to 6-flowered	(5–) 10–20-flowered	3–24-flowered
Bracteoles	linear, 3–5 mm long	linear, 3–5 mm long	linear, 6–10 mm long	linear, 2–5 mm long
Calyx	obconical, 1–2 mm long; lobes linear-lanceolate, 1.5–2 mm long	obconical, 1.2–1.5 mm long, lobes linear lanceolate, 1.6–1.8 mm long	obconical, 1–2 mm long; lobes linear, 2.5–4 mm long	obovate, 1–1.5 mm long; lobes ovate-lanceolate, 1–1.5 mm long
Corolla	white, sparsely pubescent abaxially, tubes 10–12 mm long; lobes subovate, 5–6 × 4–5 mm, glabrous inside	white, constricted at the throat, sparsely villous along the longitudinal ridge on the outside, densely yellow villous inside the throat, tubes 6–8 mm long; lobes triangular-ovate, 2–2.5 × ca. 2 mm, lobes glandular-pubescent inside	white, sparsely hairy abaxially, tubes 2–3 mm long; lobes subovate, ca. 1.5 × 1 mm, glabrous inside	white sometimes flushed with pink or purple, glabrous inside and outside; tube 25–26 mm long; lobes ovate, ca. 3.5 mm, glabrous inside
Long-styled flowers	filaments adnate at the middle of corolla tube; styles 12–15 mm long	filaments adnate to the throat of corolla tube; styles 4.5–5 mm long	filaments adnate to the base of corolla tube; styles ca. 3 mm long	filaments adnate to the throat of corolla tube; styles ca. 19 mm long
Capsule	ellipsoid, 2–3 mm, densely pubescent	ellipsoid, 1.2–2.2 mm in diam., ca. 3 mm long, densely villous	ovoid to subglobose, 3–4 mm, hairy	subglobose, 3–4 mm in diam., glabrescent

*Spiradiclis* Blume and *Ophiorrhiza* L. are two closely-related genera of the tribe Ophiorrhizeae (Rubiaceae, Rubioideae). Based on morphological characteristics, the two genera have long been recognised as near relatives and are traditionally placed in the same tribe. The taxonomic relationship between *Spiradiclis* and *Ophiorrhiza* remains historically contentious. Although Razafimandimbison and Rydin (2019) proposed synonymising *Spiradiclis* under *Ophiorrhiza*, based on molecular phylogenetics, some morphological distinctiveness in capsule architecture (linear-oblong with four valves vs. obcordate capsules with two valves) has supported traditional classification. Following the broader adoption of the molecular-based circumscription and to avoid immediate nomenclatural transfers, we place the new species in *Ophiorrhiza*. Future studies employing multi-gene phylogenetic analyses and scanning electron microscopy (SEM) of pollen morphology may help further clarify generic boundaries. Further studies integrating both molecular and morphological data and across broader sampling of species, will be necessary to fully resolve this controversy. Until then, two parallel classification systems are likely to co-exist: a phylogenetic one following Razafimandimbison and Rydin (2019) and a traditional one maintained by researchers focusing on regional floras of China and Southeast Asia. Ultimately, as molecular data become increasingly available for more species of *Ophiorrhiza*, the balance of evidence will continue to favour the expanded, monophyletic circumscription.

### Key to *Ophiorrhiza xinchengensis* and similar taxa

**Table:** 

1	Fruit strongly laterally compressed, obcordate to mitriform, dehiscing by 2 valves (sometimes further splitting, but not regularly 4-valved)	**2**
–	Fruit not compressed, subglobose to ovoid, dehiscing into 4 valves	**8**
2	Plants rooting at nodes; corolla tube less than 7 mm long	**3**
–	Plants not rooting at nodes (or rooting only at base); corolla tube 7 mm or longer	**4**
3	Corolla tube 2.5–2.8 mm; stipules 1–3 mm, caducous; fruit 5–7 mm wide	***O. pumila* Champ. ex Benth**.
–	Corolla tube 3–6 mm; stipules 4–10 mm, persistent on upper nodes; fruit 4–5 mm wide	***O. rugosa* Wall**.
4	Leaf base cordate or cordulate	**5**
–	Leaf base cuneate, attenuate, obtuse or rounded, not cordate	**7**
5	Bracts 3.5–6 mm long, well developed; leaf base regularly cordate; stems densely brown villous	***O. cordata* W.L.Sha**
–	Bracts 1–2 mm long, reduced; leaf base obtuse to cordulate; stems glabrescent or with lines of hairs	**6**
6	Leaves 1–4 × 0.6–2.5 cm; corolla tube 7–7.5 mm	***O. dulongensis* H.S.Lo**
–	Leaves 0.8–2 × 0.7–1.5 cm; corolla tube 9–10 mm	***O. kwangsiensis* Merr. ex H.L.Li**
7	Plants decumbent; stems terete, densely hirtellous; leaves 2–5 × 1–1.8 cm, both surfaces densely pubescent; stipules 1–2 mm long, linear, densely villous, usually persistent and ciliate	***O. guizhouensis* C.D.Yang & G.Q.Gou**
–	Plants erect to ascending; stems subterete to slightly compressed, glabrous or with 2 hirtellous lines; leaves variable, glabrous to sparsely pubescent; stipules usually longer (> 2 mm) or triangular	***O. japonica* Blume**
8	Plants rooting at nodes (stems prostrate, rooting at nodes)	**9**
–	Plants decumbent to ascending, not rooting at nodes (or rooting only at base)	**11**
9	Leaves 0.6–1.8 cm long, base cordulate to subtruncate; calyx lobes in fruit 2–3 × as long as capsule	***O. guangdongensis* (H.S.Lo) Razafim. & Rydin**
–	Leaves 1–5 cm long, base cuneate to rounded; calyx lobes shorter than or equal to capsule	**10**
10	Leaf blades oval to broadly ovate, 1.1–5(–6.2) × 0.5–3 cm, secondary veins 5–10 pairs; inflorescence 5–20-flowered, condensed; calyx lobes linear, 2.5–4 mm long; corolla tube 2–3 mm long (very short)	***O. longanensis* (R.J.Wang) Razafim. & Rydin**
–	Leaf blades obovate to rhombic-elliptic, 0.8–3.6 × 0.4–1.6 cm, secondary veins 4–7 pairs; inflorescence 1–4(–6)-flowered; calyx lobes linear-lanceolate, 1.6–1.8 mm long; corolla tube 6–8 mm long	***S. hechiensis* C.Xiong & L.Wu**
11	Leaves cordate to cordulate at base	**12**
–	Leaves cuneate, attenuate or obtuse at base	**13**
12	Plants creeping to ascending; leaves 1.5–4 × 1–3 cm, secondary veins 4–6 pairs; corolla tube 17–18 mm long	***O. umbelliformis* (H.S.Lo) Razafim. & Rydin**
–	Plants acaulescent or with short stems, leaves clustered at base; leaves 5–13 × 2–5.5 cm, secondary veins 15–19 pairs; corolla tube ca. 5 mm long	***O. wongiana* Razafim. & Rydin**
13	Plants acaulescent or with very short stems, leaves clustered at base; corolla tube 6–8 mm or longer	**14**
–	Plants with developed stems, leaves along distinct internodes	**15**
14	Leaves oblanceolate to obovate, 3.5–14 × 1.5–5 cm, both surfaces densely tomentose; peduncles 4.5–18 cm; corolla tube 6–8 mm	***O. robbrechtii* Razafim. & Rydin**
–	Leaves spatulate to obovate-oblanceolate, 8–13 × 2–4.5 cm, abaxially densely pubescent on veins; peduncles 7–12 cm; corolla tube 15–25 mm	***O. spathulata* (X.X.Chen & C.C.Huang) Razafim. & Rydin**
15	Corolla tube 2–3 mm long (very short)	***O. microphylla* (H.S.Lo) Razafim. & Rydin**
–	Corolla tube 10–26 mm long	**16**
16	Corolla tube 10–12 mm; leaves elliptic to oblanceolate, 2–5 cm long, densely pubescent; inflorescence 6–15-flowered	***O. xinchengensis***
–	Corolla tube 25–26 mm; leaves ovate to lanceolate, 2–9 × 1–3.3 cm, glabrous or sparsely strigillose; peduncles 0.6–2.7 cm; inflorescence 3–24-flowered; capsules subglobose, 3–4 mm in diam.	***O. scabrida* (D.Fang & D.H.Qin) Razafim. & Rydin**

## Supplementary Material

XML Treatment for Ophiorrhiza xinchengensis

## Additional information

### Artificial Intelligence (AI) use

The authors accept full responsibility for the content of the manuscript, including the disclosure of any use of AI.

No AI tools were used in the preparation of this manuscript.

### Author ORCIDs

G.-Y. Wei https://orcid.org/0000-0003-0652-1213

J.-Y. Li https://orcid.org/0000-0003-4920-4322

R. Chen https://orcid.org/0009-0006-7440-5045

Y. Nong https://orcid.org/0000-0001-7004-0946

Q.-M. Hu https://orcid.org/0009-0003-0490-3557

Y.-J. Li https://orcid.org/0009-0008-4849-2426

X.-Y. Ji https://orcid.org/0009-0002-4907-6850

C.-G. Xu https://orcid.org/0009-0000-6263-3821

Y.-K. Nong https://orcid.org/0009-0001-9097-4735

### Data availability

All of the data that support the findings of this study are available in the main text.
